# High Protein Yangyu jiaotuan (洋芋搅团): In Vitro Oral-Gastro-Small Intestinal Starch Digestion and Some Physico-Chemical, Textural, Microstructural, and Rheological Properties

**DOI:** 10.3390/foods12132460

**Published:** 2023-06-23

**Authors:** Fankui Zeng, Abhilasha Abhilasha, Yufan Chen, Yuci Zhao, Gang Liu, Lovedeep Kaur, Jaspreet Singh

**Affiliations:** 1 Lanzhou Institute of Chemical Physics, Chinese Academy of Sciences, Lanzhou 730000, China; zengfk@licp.cas.cn (F.Z.); zhaoyuci1992@163.com (Y.Z.); gangliu@licp.cas.cn (G.L.); 2 Riddet Institute and School of Food and Advanced Technology, Massey University, Palmerston North 4442, New Zealand; a.abhilasha@massey.ac.nz (A.A.); nicole.chen@donaldson.com (Y.C.); l.kaur@massey.ac.nz (L.K.)

**Keywords:** potato, Yangyu jiaotuan, microstructure, starch digestion in vitro

## Abstract

Biomimetic foods are expected to have potential health benefits for the management and prevention of chronic diseases, such as diabetes and cardiovascular disease. In the current research, two commercially available and affordable plant proteins (soy protein isolate—SPI and pea protein isolate—PPI) at two levels (5%, 10%) were added to the Yangyu jiaotuan with the objective of developing a product with reduced glycaemic properties and high protein content while maintaining its original taste and texture. The results showed that several important textural properties such as hardness and chewiness did not change significantly during the refrigerated storage. The storage modulus G′ increased with refrigerated storage time for different samples, but there were significant differences among the five samples (with and without protein addition) with respect to frequency dependence during rheological measurements. The in vitro starch digestion experiments showed that the starch hydrolysis of Yangyu jiaotuan decreased considerably (by up to 42.08%) with the increase in PPI content and during refrigerated storage due to starch retrogradation. Protein has protected the microstructure and there was less damage when compared to samples without protein. The bimodal peaks of the particle size distribution curves showed that the newly developed Yangyu jiaotuan contains two different sizes of particles; the smaller particles (~30 μm) corresponded to PPI and starch granules, while the larger particles corresponded to the fragments of the gel network of the starch matrix. Based on the above results, Yangyu jiaotuan mixed with pea protein is a convenient potato staple food product, which complies with the biomimetic potato food very well.

## 1. Introduction

One of the current and future strategies for potato processing is “Biomimetic potato foods (BPFs)” which are expected to provide excellent taste, texture, and convenience, along with superior nutritional attributes [1]. Apart from that, these foods are expected to address all food safety concerns and any issues related to environmental sustainability [2,3]. Biomimetics represents an imitation of nature’s methods, mechanisms, and processes, and it provides an inspiration to create processed BPFs with similar structure and functionality to naturally occurring healthy and nutritious foods [2]. It involves a multidisciplinary approach and considers food chemistry, food structure and digestion, food engineering and technologies, and product design [4,5]. This also helps our newly developed foods to fulfil the future expectations that the consumer is expecting from the food industry regarding food attributes.

For developing biomimetic or “natural-like” foods, it is very important to look at the structure of foods from the molecular to the macro level, and its relationship with the food’s functional and digestion-related properties [5,6]. During the consumption of foods, these structures are broken down into smaller units in the reverse order during digestion, and then they are ultimately absorbed by our body [7,8,9,10]. Apart from this, taste and texture have their importance as these are influenced by the type and level of processing [11]. The understanding and knowledge of fundamental food structure and the changes associated with it during processing and storage help us to create new dimensions for our foods.

The sticky paste-like mashed potato named Yangyu jiaotuan (洋芋搅团) is very popular in China, especially in the southeast of Gansu Province (Lintao, Wudu, and Huating). This dish uses potato as the main ingredient and is often eaten with spicy oil, vinegar, and Chinese sauerkraut. Often, the cooked mash potato is mixed vigorously and is shaped into balls for serving. Although extensive work has been carried out on starch/protein systems, very limited information is available on starch–protein interactions and their effects on microstructure, rheology, and (in vitro) starch digestibility. Alvarez et al. showed that both fresh and frozen/thawed mashed potatoes have a weak gel behaviour, without and with added SPI (soy protein isolate), together with a significant decrease in system viscoelasticity (*G*′ and *G*″) with an increasing SPI volume fraction [12]. However, to the best of our knowledge, studies involving the microstructure and digestion properties of mashed potato with SPI are scarce. 

The aim of the present study was to study some physico-chemical, textural, and rheological properties, as well as the in vitro oral-gastro-small intestinal starch digestion of Yangyyu jiaotuan. Additionally, another aim was to study the starch hydrolysis kinetics, microstructural changes, particle size distribution, and thermal properties occurring during simulated gastric and small intestinal digestion. The concept of this product was inspired by naturally occurring low glycaemic and high-protein foods, like beans and legumes. We endeavour to create interactions between starch and proteins to achieve good functionality and nutritional properties. Regarding food application, this newly-developed high-protein biomimetic potato food (BPF)—Yangyu jiaotuan—which incorporates both soy protein isolate and pea protein isolate can potentially be used as a glycaemic control potato product.

## 2. Materials and Methods

### 2.1. Materials and Sample Preparation

The potato, a total of 10 kg, was purchased from the Saturday market in Palmerston North, New Zealand. The potato variety *Solanum tuberosum* Nadine was selected due to its low solid content. After washing and peeling, the potato tubers were cut into 3 cm pieces, and boiled for 45 min. The cooked potato pieces were then mashed with a potato masher, followed by stirring at high speed for 5 min with a standard kitchen mixer. The 5% SPI or PPI Yangyu jiaotuan was made by mixing 300 g of mashed potato and 15.79 g of SPI or PPI powder in a standard kitchen mixer for 2 min. This was to form a pasta-like mashed potato. Similarly, 33.33 g protein powder was added to 300 g mashed potatoes to make the samples containing either 10% SPI or PPI. All samples were prepared in triplicate, and in order to prevent the effects of other food ingredients (spicy oil, vinegar, and Chinese sauerkraut) on the physico-chemical properties of Yangyu jiaotuan, plain mashed potato with/without protein isolates was also used in this investigation. The physico-chemical, microstructural, rheological properties, and in vitro starch digestion properties of the freshly prepared samples were analysed immediately. To investigate the effects of refrigeration on these parameters, the remaining samples with/without protein isolates were vacuum sealed and stored in a 4 °C refrigerator for 1, 3, and 5 days, respectively.

### 2.2. Textural Characteristics: Texture Profile Analysis

The textural properties of pure mashed potato (control) and samples mixed with 5% or 10% SPI and PPI were evaluated by carrying out texture profile analysis (TPA) on a Texture Analyzer (TA-XT plus, Stable Micro Systems, Godalming, UK) fitted with a load cell of 5 kg and using a flat probe (61 mm diameter). The test settings were test speed, 2 mm/s; distance, 10 mm; trigger force, 0.049 N. Samples were stored for 1, 3, and 5 days in a 4 °C refrigerator and were cut into 2 cm cubes to obtain the texture parameters of hardness, springiness, cohesiveness, gumminess, chewiness, and resilience; freshly prepared mashed potatoes were too soft to cut into pieces and thus were not tested.

### 2.3. Dynamic Rheological Properties

Dynamic oscillatory tests were conducted in an Anton Par Physica MCR301 controlled stress oscillatory rheometer (Anton Paar GmbH, Ostfildern, Germany); a geometry parallel plate with a 40 mm size was used. The temperature was maintained at (20 ± 0.1) °C, and the gap between plates was 1 mm. Frequency sweeps (from 1–100 rad s^−1^) were performed at settled stress (3–300 Pa) within the linear viscoelastic range. Dynamic rheological parameters such as storage modulus (*G*′), loss modulus (*G*″), complex modulus (G*), loss tangent (tan *δ*), complex viscosity (η*), and dynamic viscosity (*ή*) were determined for each sample as a function of frequency. Experimental data were described according to the following equations [13]:

*G*′(*ω*) = *K*′*ω^n^*′ (1)

*G*″(*ω*) = *K*″*ω^n^*″ (2)

where *G*′: a storage modulus (Pa); *G*″: loss modulus (Pa); *ω*: angular frequency (rad s^−1^); and *K*′, *K*″, *n*′, *n*″: experimental constants.

### 2.4. Starch Digestion In Vitro and Its Kinetics

A three-step digestion procedure was used, and simulated salivary fluid (SSF) containing α-amylase, simulated gastric buffer (SGF) containing pepsin, and simulated small intestine buffer (SIF) containing pancreatin, invertase, and amyloglucosidase were prepared according to [14]; the concentrations of electrolytes and enzymes besides in the simulated digestive fluids were similar to the literature [15].

SSF (pH 7) stock was prepared by adding 2.1 g NaHCO_3_, 0.117 g NaCl, and 0.14 g KCl to 250 mL d^2^ H_2_O, and was split into small quantities and stored in a freezer. SGF (pH 1.2) stock was prepared by adding 2 g NaCl, 7 mL 12 M HCl to 800 mL d^2^ H_2_O, and filling it up to 1 L. SIF (pH 6.8) stock was prepared by adding 6.8 g monobasic potassium phosphate (KH_2_PO_4_) to 250 mL H_2_O, then 77 mL 0.2 N NaOH was added, pH-adjusted with NaOH/HCl to 6.8, filled up to 1 L with d^2^ H_2_O, and stored in a 4 °C fridge.

SSF (pH 7) containing α-amylase (2.0 g/L) was prepared fresh, and, before use, one portion of concentrated SSF with three portions of d^2^ H_2_O was added to it. SGF containing pepsin (enzyme/starch (dry weight basis) ratio, 1.765:100, *w*/*w*) was prepared fresh, by adding 0.24 g of pepsin (4 °C, 800–2500 units/mg protein) to 50 mL gastric buffer, and covering and stirring for 10 min at 4 °C. SIF containing pancreatin (enzyme/starch (dry weight basis) ratio, 1.3:100, *w*/*w*), amyloglucosidase (enzyme/starch (dry weight basis) ratio, 0.26:1, *v*/*w*), and invertase (enzyme/starch (dry weight basis) ratio, 1:1000, *w*/*w*) was prepared fresh by adding 0.2 g pancreatin (−20 °C, 4 UPS), 15 mg invertase (−20 °C, Grade VII from baker’s yeast 401 U/mg solid), 4 mL amyloglucosidase (4 °C, Megazyme 3260 U/mL), 46 mL intestinal buffer, and was covered and stirred for 10 min at 4 °C. The starch content of the Nadine potato variety (63.9% db) and Yangyu jiaotuan with/without the protein isolates were measured using a Megazyme Total Starch Assay Kit (AA/AMG), and 100 assays.

SSF, SGF, and SIF were re-warmed to 37 °C before use. For the oral digestion, the Yangyu jiaotuan sample contained 6.8 g starch, mixed with SSF at a mass ratio of 1:1, and topped up to 170 g with distilled water. After 2 min of oral digestion, the pH was adjusted to 2 using 3 M and 0.5 M HCL, and then 25 mL SGF was added to start the gastric digestion. After 30 min, the pH of the digestion system was adjusted to 6.8 using 3 M and 0.5 M NaOH, followed by adding 23 mL SIF to start the small intestinal digestion. Both the volume of HCL and NaOH were recorded for calculating the starch hydrolysis (%).

Starch digestibility was measured using the glucose released after a certain time of simulated digestion. The glucose released was analysed using a GOPOD reagent (Format K-GLUK 07/11, Megazyme International Ireland Ltd., Wicklow, Ireland) and the results were expressed as starch hydrolysis (%) [16]. In total, 0.5 mL samples were taken during the digestion procedure, and added to 2 mL of 96% ethanol to stop the digestion; O_2_ refers to 2 min of oral mastication; G_0_, G_15_, and G_30_ refer to 0, 15, and 30 min of gastric digestion; I_0_, I_5_, I_10_, I_15_, I_30_, I_90_, and I_120_ correspond to 0, 5, 10, 15, 30, 90, and 120 min of small intestinal digestion. The hydrolysis index (HI) and estimated glycaemic index (*e*GI) of the samples were calculated according to the reported method [17], using white bread as a reference.

### 2.5. Microstructural Characteristics

#### 2.5.1. Light Microscopy (LM)

Freshly-prepared pure mashed potato and samples mixed with 5% or 10% pea proteins were examined with a Leica CME light microscope (Leica Microsystems GmbH, Wetzlar, Germany); the magnifying power was 100×, samples were stained with 1% iodine indicator, and the mashed potato microscopy images were processed using the OMAX ToupView V3.7 software.

#### 2.5.2. Scanning Electron Microscopy (SEM)

SEM analysis was performed to examine the topographic characteristics of washed and freeze-dried mashed potato particles coated with gold (SCD 050, Balzers, Liechtenstein, Germany); an FEI Quanta 200 FEI Electron Optics scanning electron microscope (Eindhoven, The Netherlands) was used to examine the samples. Approximately 100 g fresh prepared mashed potato was added to a 1000 mL beaker, 800 mL of ultrapure water was added, the sample was gently stirred and dispersed with a glass rod, poured when the mashed potato particles were settled (approximately stand for 10 min), and then 800 mL of water was added and the cleaning was repeated twice. The sediment was collected in a zip plastic bag, immediately immersed in liquid nitrogen for quick freezing, and then freeze-dried (Cuddon FD18 Freeze Drier (model), Cuddon Freeze Dry (Manufacturer), Blenheim, New Zealand) to obtain the mashed potato particles. The frozen samples were loaded in at approximately −20 °C (shelf temperature). The condenser setting was −40 °C. Once the vacuum was sufficient, approximately 1 millibar, the shelf temperature was adjusted to 20 °C to speed up the sublimation process. One drying run was approximately 72 h long. Yangyu jiaotuan digesta was also examined using SEM, and the same experimental condition was used.

#### 2.5.3. Confocal Laser Scanning Microscopy (CLSM)

The pure mashed potato (control) and samples mixed with 5% or 10% pea proteins were immediately transferred onto Labserv microscope slides (Thermo Fisher Scientific, Waltham, MA, USA) for CLSM analysis; samples were stained with the fluorescent dye Fast green [0.1% (*w*/*v*)] and fluorescein isothiocyanate (FITC) [0.01% (*w*/*v*)] for 20 min. Then, the stained sections were observed with a TCS SP5 DM6000B Laser Scanning Confocal Microscope (Leica Microsystems, Wetzlar, Germany). The excitation wavelength of the laser was 561 nm, while the emission wavelength was set to 517 nm for potato starch and 575 nm for pea protein. The samples were loaded on a motorized XY stage, and digital image files were recorded at a resolution of 1024 × 1024 pixels.

### 2.6. Particle Size Distribution

The particle size distribution of Yangyu jiaotuan digesta at different digestion stages was determined according to the reported method [14], using laser diffraction particle size analysis (Mastersizer 2000; Malvern Instruments Ltd., Malvern, UK). The relative refractive index applied was 1.70.

### 2.7. Thermal Analysis

The thermal properties of the Yangyu jiaotuan digesta were determined using differential scanning calorimetry (DSC) (DSC; TA Q100, TA Instruments, Newcastle, DE, USA). Samples (approximately 5.0 mg, dry basis) were weighed directly in an aluminium pan, and distilled water was added to obtain a starch–water ratio of 1:3 (*w*/*w*). The pan was hermetically sealed and allowed to equilibrate for 1 h before analysis. The sample pans were then heated from 20 to 100 °C at a rate of 5 °C/min. An empty pan was used as a reference.

### 2.8. Statistical Analysis

The results are expressed as means ± standard deviation (*n* = 3). Subsequently, an analysis of variance (ANOVA) with Tukey’s test was used to determine significant differences among the means at a significance level of *p* < 0.05. The data were subjected to correlation analysis, and Pearson correlation coefficients were calculated using IBM SPSS Statistics (Version 22) Software version 13 (Minitab Inc., State College, PA, USA).

## 3. Results and Discussion

### 3.1. Texture Profile Analysis and Textural Characteristics

**Figure 1:**
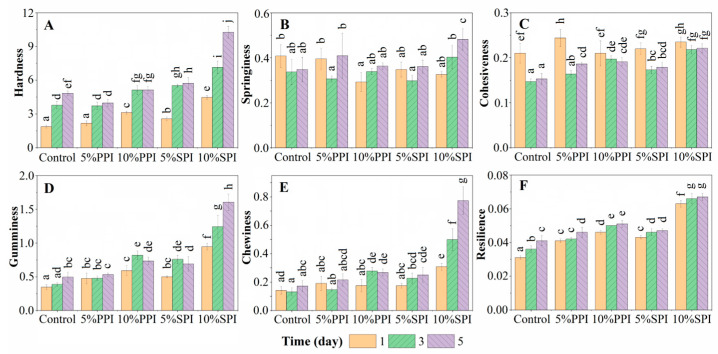
Effect of refrigerated storage on the textural parameters of Yangyu jiaotuan, different letters indicate significant difference at the significance level of *p* < 0.05. (**A**) Hardness; (**B**) springiness; (**C**) cohesiveness; (**D**) gumminess; (**E**) chewiness; (**F**) resilience.

The textural parameters of the Yangyu jiaotuan, like hardness, springiness, cohesiveness, gumminess, chewiness, and resilience, determined using texture profile analysis (TPA) are presented in Figure 1. The TPA test was conducted by subjecting Yangyu jiaotuan to a compressive deformation followed by a relaxation and then a second compressive deformation [18]. The hardness, gumminess, chewiness, and resilience increased with the rising content of PPI and SPI in Yangyu jiaotuan (Figure 1). The changes in those parameters may be attributed to the low moisture content in Yangyu jiaotuan made with the addition of PPI and SPI, as the moisture content of Nadine potato tuber was 89.31% ± 0.17, while the values for PPI and SPI were 5.44% ± 0.23 and 6.45% ± 0.39, respectively. The weight of potato pieces increased slightly (2.4%) after cooking, and the moisture contents of the control samples, 5% PPI, 10% PPI, 5% SPI, and 10% SPI were 89.56% ± 0.65, 84.20% ± 0.51, 80.05% ± 0.32, 85.41% ± 0.52, and 81.25% ± 0.49, respectively.

**Figure 2:**
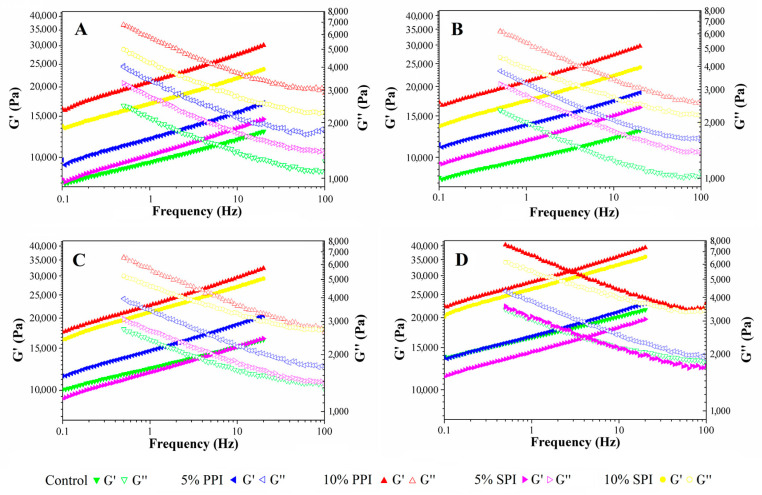
Effect of refrigerated storage on the dynamic rheological properties storage modulus (G′), and loss modulus (G′′) of Yangyu jiaotuan. (**A**) Freshly prepared; (**B**) 1 day of refrigerated storage; (**C**) 3 days of refrigerated storage; (**D**) 5 days of refrigerated storage.

Springiness (Figure 2B) values varied from 0.29 for Yangyu jiaotuan made with 10% PPI to 0.48 for Yangyu jiaotuan made with 10% SPI, while the cohesiveness (Figure 2C) decreased with the addition of both PPI and SPI. The results showed that several important textural properties such as hardness and chewiness did not change significantly during the refrigerated storage. Figure 1 also shows that SPI at a high concentration (10%) has a great impact on the hardness, gumminess, and chewiness of the sample; this may be due to SPI’s strong ability to absorb water, which cannot be reflected at a relatively low concentration (5%). The extent of change in the texture of Yangyu jiaotuan was subtle in the samples containing PPI, reflecting its suitability for use in this product.

### 3.2. Rheological Properties

The dynamic rheological properties, storage modulus (*G*′) and loss modulus (*G*″), are presented as a function of the frequency in Figure 2 and Table 1; there were significant differences among the five mashed potato samples (with and without protein) with respect to frequency dependence during rheological measurements. In each case, the modulus versus frequency curves for the different samples exhibited no cross-over within the range of frequency accessed (Figure 2); *G*′, *G*″ and complex (G*) increased while complex viscosity (η*) and dynamic viscosity (*ή*) decreased with increasing frequency. All the values of rheological parameters *G*′, *G*″, G*, loss tangent (tan δ), and η* and *ή* increased with the addition of both PPI and SPI in the Yangyu jiaotuan, and the values increased with the increasing PPI and SPI content, except for the loss tangent.

The *G*′ of the control on the day zero was about 12,000 Pa; it remained the same on the first day, then increased to about 17,000 Pa on the third day, and increased to about 22,000 Pa on the fifth day, which is due to starch retrogradation during the storage. The values of Yangyu jiaotuan made with 5% plant proteins (both PPI and SPI) were close to the control at day zero, and during the storage times of 1 day, 3 days, and 5 days, it had very similar characteristics to the control. At the end, even on the fifth day, the Yangyu jiaotuan made with 5% SPI addition had the lowest viscosity values among all. It was even lower than the control. The values of rheological parameters increased significantly when 5%PPI, 10%PPI, or 10% SPI was added. Yangyu jiaotuan made with 10% PPI had the highest viscosity and other rheological parameters; it was very different from the control, indicating a very strong gel.

Rheological techniques can also be used to monitor the development of the viscoelastic properties of starch during retrogradation [19]. In this study, during the refrigerated storage at 4 °C, the values of *G*′, G*, and η* increased significantly with the prolongation of storage time from 0 days to 5 days, while the values of *G*″, and *ή* did not change significantly. The values of tan *δ* decreased, and exhibited behaviour contrary to *G*′, *G*″, G*, η*, and *ή*, indicating the formation of stronger network structures in retrograded pastes.

Table 2 illustrates the extent of change in *G*′, *G*″, and η* by percentage. For the control sample, the value of *G*′ after 5 days increased by 77.27%, while it was lower for the sample containing 5% PPI, showing a 45% increase. The extent of change in *G*″ and η* in terms of percentage shows a similar pattern to the *G*′ values; this means that the protein has helped the sample to prevent starch retrogradation. Usually, consumers like to eat soft food; so, we can keep Yangyu jiaotuan soft during the storage period by adding plant proteins. Moreover, the value of *G*′ after five days increased by 29.46% and 53.35% for the Yangyu jiaotuan containing 10% PPI and 10% SPI, respectively, which indicated that with high extra protein addition, PPI is more effective in preventing starch retrogradation and ultimately obtaining a smoother or softer consistency over the storage period. On the other hand, during storage, water migrates slowly from the starch matrix to the protein granules [12], thus increasing the storage resistance of protein-rich mashed potatoes. Noh et al. [20] reported that low-temperature treatment increased the hydrophobicity of soy protein regardless of the heating treatment. Alvarez et al. [12] reported that a freeze/thaw cycle produced a significant decrease in the viscoelastic functions of mashed potato enriched with SPI, which is due to the superior aggregation of denatured SPI and reduced water activity.

Although the rheological parameters have already given a lot of information about how PPI and SPI affect starch retrogradation, further studies are required to better understand the mechanism behind it. There are plenty of works that have already been conducted on starch/protein systems, but very little is reported in them [12]. It has been reported that potato and rice flour can be used to partially replace wheat flour in bread processing [21,22], and that the rheological properties of dough can be kept unchanged.

The Pearson correlation coefficients for the relationships between textural and rheological properties are presented in Table 3. Hardness, gumminess, chewiness and resilience showed a positive correlation with *G*′ (*r* = 0.658, 0.680, 0.659, 0.692, respectively), and rheological properties such as G′, G″, and peak viscosity also showed positive correlations (*r* = 0.690, 0.847, 0.736, respectively).

The correlation between these texture parameters and *G*″ and G* shows the same pattern because gumminess, chewiness, and resilience showed a strong positive correlation with hardness, while *G*″, G*, η*, and *ή* also had a strong positive correlation with *G*′.

### 3.3. Starch Hydrolysis (%) and Estimated Glycaemic Index

**Figure 3:**
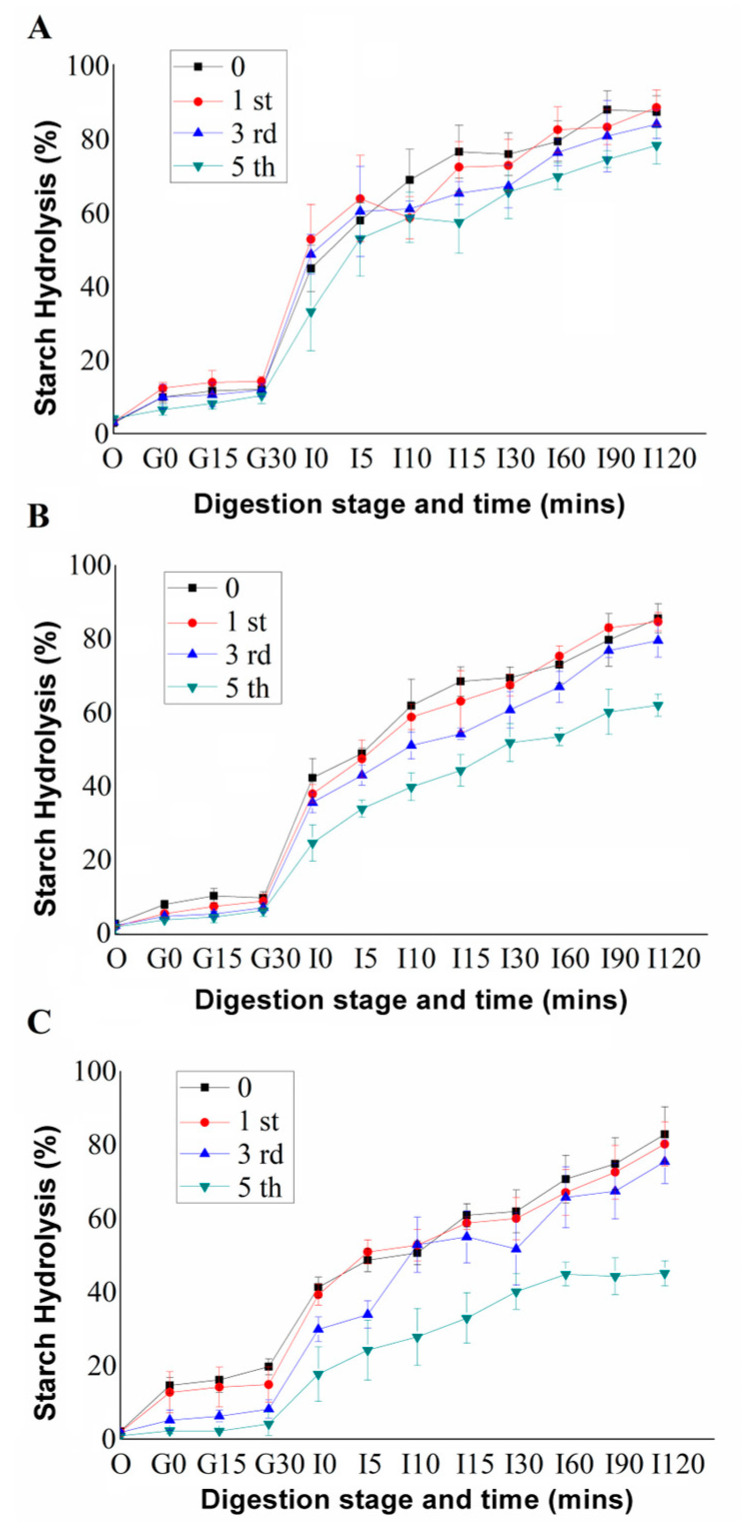
Starch hydrolysis (%) of plain Yangyu jiaotuan (**A**), mixed with 5% PPI (**B**), and 10% PPI (**C**) after refrigerated storage at 4 °C for 0, 1, 3, and 5 days. SEM images of washed Yangyu jiaotuan.

The starch hydrolysis (%) of Yangyu jiaotuan during in vitro oral-gastro-small intestinal digestion is shown in Figure 3. During the simulated oral digestion phase (O and G_0_), α-Amylase is generally well integrated within the food bolus and continues to release some glucose; the starch hydrolysis (%) observed during the oral digestion process ranged from 2.27% to 14.60%. During the simulated gastric digestion phase (G_0_ and G_30_), the starch hydrolysis (%) of all samples was barely changed, indicating that the activity of α-amylase is very low due to the extremely low pH of 2 (Figure 3). During the simulated small intestinal digestion, the starch hydrolysis (%) of all samples increased rapidly, especially at the initial phase. From Figure 3, it was obvious that the starch hydrolysis (%) of all samples decreased with the extension of refrigerated storage time from 0 days to the 5th day. The gradually decreasing starch hydrolysis (%) at 0 days (76.34%), 1 day (72.17%), 3 days (65.09%), and 5 days (57.19%) of the plain Yangyu jiaotuan (control) at the I15 point indicated that refrigerated storage resulted in the starch retrogradation process, and, subsequently, the rate of enzymatic digestion, which is consistent with our previous study [14].

The equilibrium starch hydrolysis percentage (C_∞_), areas under hydrolysis curves (AUC), hydrolysis index (HI), and estimated glycaemic index (*e*GI) were estimated by fitting a first-order equation model according to the reported method [17]. The HI of the freshly-prepared plain Yangyu jiaotuan was 129.84, while the white bread was used as a reference. The HI and the *e*GI of all the samples decreased with increasing refrigerated storage, indicating that chilled storage affected the rate of enzymatic digestion (Table 4). The experimental C_∞_ value of 5% PPI and 10% PPI samples after 5 days of refrigerated storage was significantly lower than the values of other samples, while the differences in the values of other samples were not significant; undoubtedly, the starch retrogradation played a dominant role. For the same refrigerated storage time of 0, 1, 3, and 5 days, the higher the amount of protein added, the lower the HI and *e*GI values. The 10% PPI after 5 days of refrigerated storage had the lowest HI (64.36) and *e*GI (75.04) values, which is significantly lower than the other values of the control and 5% PPI. Factors that influence the kinetics of starch digestion are the nature of the starch, physical form, protein and lipid interactions, the presence of antinutrient enzyme inhibitors, and food processing [23]. The results in this study showed that both the addition of extra plant protein and the starch retrogradation during refrigeration could slow down the enzymatic hydrolysis of starch and reduce *e*GI in the Yangyu jiaotuan.

Figure 3 and Table 4 clearly show that the newly developed products with extra plant proteins have a lower starch hydrolysis or estimated glycaemic index than the control sample, implying that the protein granules could act as a physical barrier, impeding amylase accessibility to the starch. The protein appeared to hinder the free access of amylolytic enzymes to the starch, therefore providing a slow and extended-release of glucose during in vitro digestion. This means that compared with plain mashed potato, Yangyu jiaotuan with high protein content is resistant to amylase digestion and can release glucose for energy for a longer period of time.

The ultimate vision of this study is not to make a replica of natural foods, but rather to be inspired by the structure–functionality linkages encoded in them, and to enable the design of nature-like food systems with similar or enhanced functionality [2]. Scientific evidence has suggested that intact, minimally-processed, natural foods seem to have more compact, cohesive matrices and stronger interactions between nutrients [1]. We have developed a category of BPF products of high-protein Yangyu jiaotuan that can be tailored to allow their use in specific health-promoting applications. These may include applications to prevent or combat chronic diseases, as its reduced caloric content and prolonged satiety can help with obesity management, and its slow and sustained release of glucose in the small intestine and delivery of resistant starch to the large intestine can decrease the risk of diabetes and improve colonic health.

### 3.4. Microstructural Characteristics

**Figure 4:**
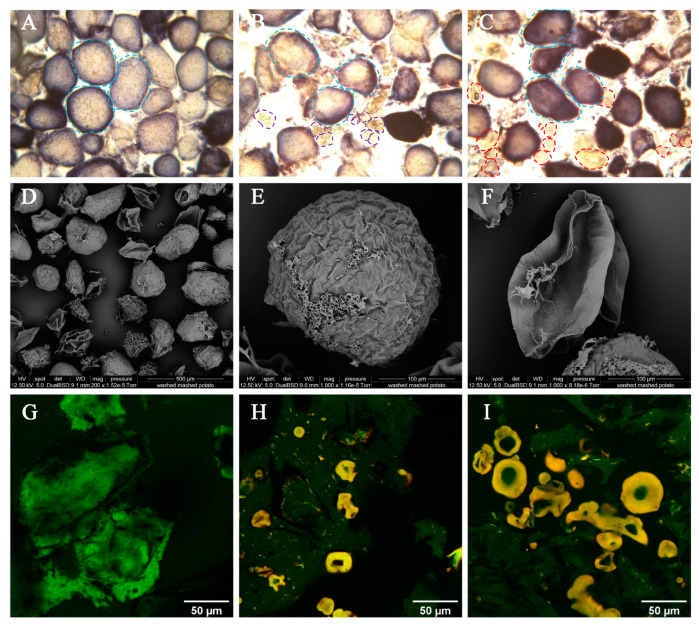
Light micrographs (100×) of Yangyu jiaotuan ((**A**) control; (**B**) with 5% SPI; (**C**) with 5% PPI); the blue dashed circle refers to starch matrix; the purple dotted circle refers to SPI; the red dotted circle refers to PPI. SEM images of washed Yangyu jiaotuan ((**D**) 200×; (**E**,**F**) 1000×) the CLSM time-lapse images of fresh prepared Yangyu jiaotuan stained by fast green and FITC ((**G**) plain Yangyu jiaotuan; (**H**) with 5% PPI; (**I**) with 10% PPI).

Microphotographs of Yangyu jiaotuan are shown in Figure 4. As shown in the microphotographs of a light microscope (Figure 4A–C), large size particulars in blue and small size particulars in gold can be distinguished, and they refer to starch (stained by 1% iodine indicator) and globular protein, respectively. A fair amount of golden granules can be found in the Yangyu jiaotuan made with 5% PPI (Figure 4B) and 5% SPI (Figure 4C); the morphology characteristics, such as colour and shape, were very similar, but the particle size of SPI was relatively higher than that of PPI.

Alvarez et al. [12] reported that two different types of gel networks, fine-stranded and coarse networks, were formed in mashed potatoes enriched with SPI. In our study, two distinct particles were found, the large particles of a blue dashed circled starch matrix (Figure 4B,C), and small particles of protein (Figure 4B, purple dotted circled SPI, Figure 4C, red dotted circled PPI). The absence of adhesion between the starch matrix (Figure 4A–C, blue dashed circled) should be disregarded, as this is not a feature of the different samples but a problem with sample preparation, as the samples were dispersed and stained with a 1% iodine indicator before the light microscope examination.

A strong gel network of the starch matrix was formed in Yangyu jiaotuan, which can be confirmed by the results of the CLSM images (Figure 4G–I). The opaque, coarse gel network, defined as the green aggregate (stained by Fast green), was made with a starch matrix (Figure 4G). The yellow protein globules (Figure 4H,I) are evenly dispersed in the starch matrix, indicating that Yangyu jiaotuan (over mashed potato) mainly consists of a continuous amylose/amylopectin matrix phase due to the gelatinization of the potato starch granules following heating and vigorous stirring.

Globular protein granules can be found in Figure 4B (purple dotted circled SPI) and Figure 4C (red dotted circled PPI), which are distinguishable from the starch matrix. The absence of adhesion between protein granules can also be found in the CLSM time-lapse images of freshly-prepared Yangyu jiaotuan (Figure 4H,I), indicating that the protein gel was not formed. Turgeon and Beaulieu [24] reported that the main factors affecting the formation and properties of protein gel include environmental conditions, protein composition, denaturation degree and concentration, and processing conditions, etc. Tseng et al. observed that SPI gels exhibited a particulate porous network structure [25]; nevertheless, when the SPI network structure becomes coarser, the ability of the gels to retain water decreases [26]. 

To achieve a better understanding of the starch matrix of Yangyu jiaotuan, freshly-prepared Yangyu jiaotuan was washed with plenty of water, followed by freeze-drying; the microstructure of the systems was studied by SEM (Figure 4D–F). Individual cells were successfully isolated and captured, and the size of freeze-dried potato cells ranged from 100 to 200 microns, which is much smaller than the potato parenchyma cells that ranged from 100 to 1000 microns [11]. The starch matrix is tightly packed within the cell wall polymer network, and the folds on the surface of microspheres can explain the dehydrating effect of freeze-drying on the shrinking cell volume. Micrographs also revealed the presence of cell wall cementing materials, as well as cell fragments, being embedded in the continuous solubilized starchy matrix (Figure 4D,F), which was consistent with a previous report [12]. The native starch granules absorbed abundant cell fluid and gelatinized during hydrothermal cooking, followed by the separation of intact cells (instead of rupturing) during Yangyu jiaotuan processing due to the drastic mechanical shearing. Once there has been a substantial level of cell separation, it becomes rather difficult to fracture individual cells by any mechanical means [25,27]. Berg, Singh, Hardacre et al. and Tydeman et al. also reported that the hydrothermal processing of plant foods (e.g., legumes and carrots) and subsequent mechanical shear applied within the resulting puree induce the separation of intact cells without breaking them open [28,29].

**Figure 5:**
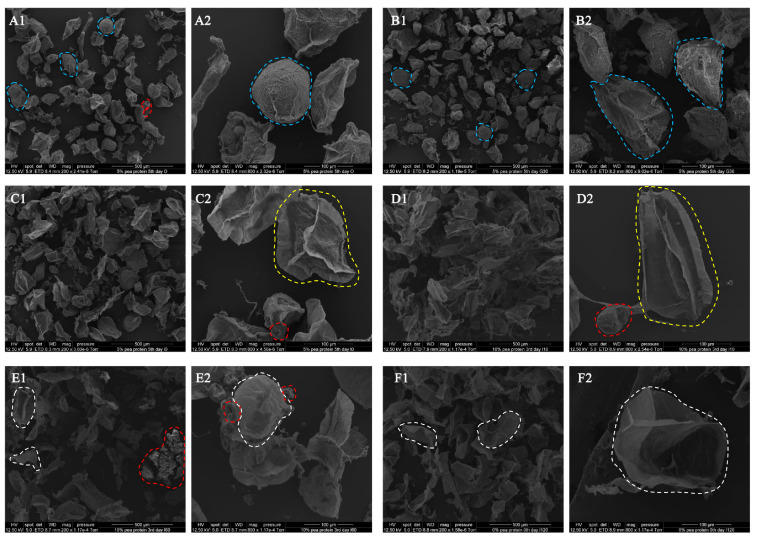
SEM images of Yangyu jiaotuan digesta with 5% pea protein after 5-day refrigerated storage at O_2_ (**A**), 5% pea protein after 5-day refrigerated storage at G_30_ (**B**), 5% pea protein after 5-day refrigerated storage at I_0_ (**C**), 10% pea protein after 3-day refrigerated storage at I_10_ (**D**), 10% pea protein after 3-day refrigerated storage at I_60_ (**E**), and fresh prepared plain mashed potato at I_120_ (**F**). The blue dashed circle refers to starch matrix, while the red dotted circle refers to PPI. The yellow dotted circles refer to the starch matrix in which part of the starch has been digested, and the white dotted circle refers to the cell wall polymer.

During the simulated oral digestion and simulated gastric-small intestinal digestion, the microstructural changes of mashed potato mixed with pea protein were also revealed via SEM (Figure 5). After 2 min of oral digestion (Figure 5A), the morphology of the starchy matrix did not change compared with that of freshly prepared Yangyu jiaotuan (Figure 4D,E). Similarly, there was no obvious change after 30 min of simulated stomach digestion (Figure 5B); this observation was attributed to the absence of amylases in the gastric juice, with the minimal hydrolysis observed being attributed to acid pH [30]. During the initial phase of simulating small intestine digestion, the starchy matrix shrank to become smaller, and the cell wall polymer network appeared (Figure 5C). After 10 min of simulated small intestine digestion, a large number of cell-wall polymer network fragments are seen (Figure 5D). As the digestion process progresses, the starch matrixes are nearly invisible, leaving only the cell wall polymer network fragments and the pea protein particles (Figure 5E). In vitro starch digestion assays combined with SEM techniques demonstrated that gelatinized starch, which is tightly packed within biopolymer matrices in cooked starch matrixes, was enzyme-digested in a layer-by-layer fashion (Figure 5C2); this means that digestion progresses from the outer part towards the centre of the starch-entrapped microspheres [1]. Figure 5F is a plain mashed potato without additional plant protein. No starch matrix could be found after 120 min of simulated small intestine digestion, and all that remained were cell wall polymer networks that could not be digested by amylase.

### 3.5. Particle Size Distribution and DSC Thermograms of Yangyu jiaotuan Digesta

**Figure 6:**
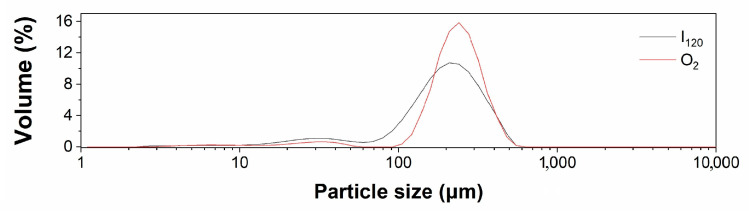
Particle size distribution for Yangyu jiaotuan digesta with 5% PPI after 3 days of refrigerated storage.

The particle size distributions for Yangyu jiaotuan digesta with 5% PPI after 3 days of refrigerated storage are shown in Figure 6. The bimodal peaks of the particle size distribution curves show that the newly developed Yangyu jiaotuan contains two different sizes of particles; the smaller particles corresponded to PPI (~30 μm), which is consistent with the CLSM and SEM results (Figure 4I and Figure 5E2). Our previous study revealed that plain cooked potato tuber after simulated digestion contains some small particles sized between 15.2 and 35.1 μm [14]. Those small particles corresponding to starch granules can also be found in the Yangyu jiaotuan. However, the volume of large particles present in Yangyu jiaotuan (~10%) was much greater than that presented in plain cooked potato (0.5–1.5%). The larger particles in Yangyu jiaotuan corresponded to the fragments of a gel network of the starch matrix [31], which is consistent with the SEM results (Figure 5). The results also show that the integrated volume of the small particle sizes increased, and the volume of the large particle sizes decreased as the digestion time was extended, indicating that the fragments of the starch matrix became smaller and smaller during prolonged digestion.

**Figure 7:**
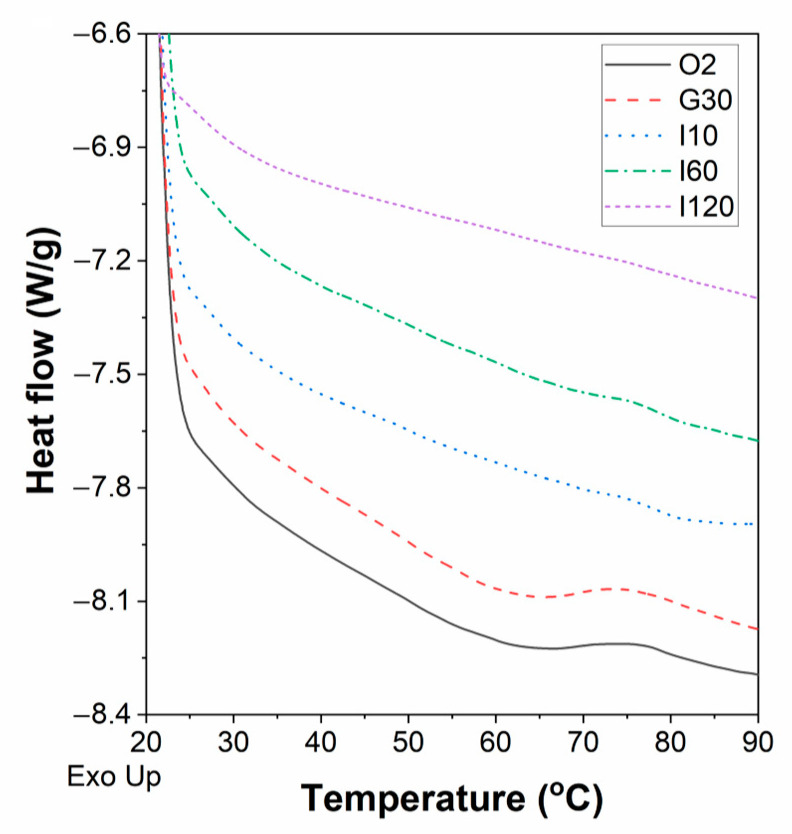
Differential scanning calorimetry (DSC) thermogram of Yangyu jiaotuan digesta with 5% PPI after 5 days of refrigerated storage during the simulated oral-gastric-small intestinal digestion.

The results for the differential scanning calorimetry (DSC) thermogram of Yangyu jiaotuan digesta samples during the simulated oral-gastric-small intestinal digestion are shown in Figure 7. The peak temperatures (*T*_p_) of O_2_ and G_30_ were found to be 75.47 and 75.75 °C, respectively; the intensity of exothermic peak in samples decreased gradually as the simulated digestion time extended, and the absence of significant exothermic peaks in I_10_ and I_60_ samples indicates that most of the potato starch has been digested. At the end of the simulated small intestinal digestion, no exothermic peak could be found, which indicates that there is no starch matrix residue in the sample I_120_; these results were consistent with those of starch hydrolysis (Figure 3) and SEM (Figure 5).

## 4. Conclusions

The design and manufacturing of the high-protein, low-glycaemic, biomimetic potato food Yangyu have broad commercialization, and the prospects are very exciting. Although potato tubers already contain high-quality protein, adding extra plant proteins (soy protein isolate—SPI and pea protein isolate—PPI) as nutrient enhancers to the Yangyu jiaotuan will have many benefits: firstly, the glycaemic index can be reduced; secondly, the protein content can be increased; thirdly, the product can maintain the same taste and texture; fourthly, the price is affordable; fifthly, it is a convenience food; finally, such a potato product is a staple food for its consumer base. The organoleptic properties of Yangyu jiaotuan, the biomimetic potato food in this research, were studied. The research showed that several important textural properties such as hardness and chewiness did not change significantly during the refrigerated storage. Yangyu jiaotuan was fortified with different proteins at different levels, and the results demonstrate that the addition of pea protein isolate enhances the texture profile compared to the addition of soy protein isolate. The in vitro starch digestion experiments showed that the starch hydrolysis of Yangyu jiaotuan decreased considerably with the increase in PPI content and during refrigerated storage due to starch retrogradation. In addition, the particle size distribution analysis experiments showed that the developed Yangyu jiaotuan contains two different sizes of particles; the smaller particles corresponded to PPI and starch granules, while the larger particles corresponded to the fragments of the gel network of the starch matrix.

Based on the popularity of this potato-based product in China, this product was selected to create a high protein and low glycaemic version of Yangyu jiaotuan. This project can be very useful for the potato processing industry in China if it decides to manufacture this new Yangyu jiaotuan, which is a very healthy and nutritious food, especially for people with diabetes and obesity. The starch hydrolysis during in vitro gastro-small intestinal digestion clearly shows that the newly developed product has a lower starch hydrolysis or estimated glycaemic index than the control sample. It provides more protein and a lower glycaemic index, and is a convenience food that fits the modern lifestyle and the demands of the millennial population in China. We expect that it will be a big success if this product reaches the restaurant market in China. This product also fits with the Chinese Government’s policy of “Potato as a staple food” and complies with the biomimetic potato food very well.

## Figures and Tables

**Table 1:** Rheological properties of Yangyu jiaotuan.

Sample	Time (Day)	Rheological Properties during Frequency Sweep ^a^					
		*G′* (Pa)	*G*″ (Pa)	G* (Pa)	tan *δ*	η* (Pa s)	*ή* (Pa s)
Control	0	12,406.67 ± 601.86 ^a^	2451.33 ± 98.35 ^a^	12,646.67 ± 608.80 ^a^	0.20 ± 0.00 ^hi^	100.650 ± 4.84 ^a^	19.50 ± 0.78 ^a^
	1	12,670.00 ± 357.63 ^a^	2263.33 ± 64.47 ^a^	12,870.00 ± 361.66 ^a^	0.18 ± 0.00 ^d^	102.423 ± 2.88 ^a^	18.04 ± 0.48 ^a^
	3	17,130.00 ± 742.23 ^cd^	2809.67 ± 75.58 ^b^	17,353.33 ± 744.47 ^c^	0.16 ± 0.008 ^b^	138.100 ± 5.93 ^c^	22.36 ± 0.60 ^b^
	5	21,993.33 ± 349.33 ^f^	3518.33 ± 57.93 ^de^	22,273.33 ± 349.33 ^e^	0.16 ± 0.00 ^a^	177.233 ± 2.80 ^e^	28.00 ± 0.47 ^de^
5% PPI	0	15,983.33 ± 1485.98 ^c^	3800.33 ± 305.35 ^e^	16,426.67 ± 1514.48 ^c^	0.24 ± 0.00 ^m^	130.733 ± 12.04 ^c^	30.24 ± 2.43 ^e^
	1	18,786.67 ± 85.05 ^de^	3782.67 ± 4.73 ^e^	19,160.00 ± 80.00 ^d^	0.20 ± 0.00 ^i^	152.467 ± 0.65 ^d^	30.10 ± 0.04 ^e^
	3	19,720.00 ± 713.58 ^e^	3797.00 ± 139.93 ^e^	20,056.67 ± 685.30 ^d^	0.19 ± 0.00 ^g^	159.833 ± 5.75 ^d^	30.22 ± 1.12 ^e^
	5	23,183.33 ± 242.14 ^fg^	4339.33 ± 65.455 ^f^	23,583.33 ± 250.07 ^ef^	0.19 ± 0.00 ^ef^	187.667 ± 1.95 ^ef^	34.53 ± 0.52 ^f^
10% PPI	0	29,863.33 ± 814.51 ^hi^	6837.67 ± 172.93 ^j^	30,636.67 ± 829.84 ^gh^	0.23 ± 0.00 ^l^	243.800 ± 6.56 ^gh^	54.42 ± 1.38 ^j^
	1	28,710.00 ± 814.13 ^h^	5971.00 ± 152.97 ^h^	29,326.67 ± 825.67 ^g^	0.21 ± 0.00 ^j^	233.367 ± 6.61 ^g^	47.52 ± 1.22 ^h^
	3	31,116.67 ± 1001.67 ^i^	6269.67 ± 190.08 ^i^	31,740.00 ± 1024.31 ^h^	0.20 ± 0.00 ^i^	252.567 ± 8.15 ^h^	49.90 ± 1.51 ^i^
	5	38,660.00 ± 606.05 ^j^	7502.67 ± 104.01 ^k^	39,383.33 ± 611.58 ^i^	0.19 ± 0.00 ^gh^	313.400 ± 4.8816 ^i^	59.70 ± 0.83 ^k^
5% SPI	0	14,190.00 ± 1212.97 ^b^	3191.67 ± 245.90 ^c^	14,543.33 ± 1241.02 ^b^	0.23 ± 0.00 ^k^	115.767 ± 9.86 ^b^	25.40 ± 1.96 ^c^
	1	15,786.67 ± 440.72 ^c^	3148.67 ± 59.69 ^c^	16,100.00 ± 446.43 ^c^	0.20 ± 0.00 ^i^	128.100 ± 3.59 ^c^	25.06 ± 0.48 ^c^
	3	16,466.67 ± 775.13 ^c^	3106.33 ± 125.60 ^c^	16,756.67 ± 785.13 ^c^	0.19 ± 0.00 ^f^	133.367 ± 6.25 ^c^	24.72 ± 1.00 ^c^
	5	18,473.33 ± 1115.05 ^de^	3387.67 ± 148.08 ^cd^	18,783.33 ± 1124.47 ^d^	0.18 ± 0.00 ^e^	149.467 ± 8.92 ^d^	26.96 ± 1.18 ^cd^
10% SPI	0	24,560.00 ± 919.95 ^g^	5169.67 ± 202.44 ^g^	25,103.33 ± 944.79 ^f^	0.21 ± 0.00 ^j^	199.733 ± 7.4782 ^f^	41.14 ± 1.61 ^g^
	1	24,710.00 ± 547.81 ^g^	4554.67 ± 69.21 ^f^	25,126.67 ± 553.38 ^f^	0.18 ± 0.00 ^ef^	199.967 ± 4.36 ^f^	36.24 ± 0.55 ^f^
	3	29,616.67 ± 368.56 ^hi^	5263.00 ± 48.54 ^g^	30,080.00 ± 370.41 ^gh^	0.18 ± 0.00 ^d^	239.367 ± 2.97 ^gh^	41.88 ± 0.39 ^g^
	5	37,663.33 ± 1652.58 ^j^	6416.67 ± 276.31 ^i^	38,210.00 ± 1677.71 ^i^	0.17 ± 0.00 ^c^	304.033 ± 13.32 ^i^	51.06 ± 2.20 ^i^

^a–m^ Values with the same superscript in a column did not differ significantly (*p* < 0.05); results are expressed as means ± standard deviation (*n* = 3), and rheological property values at 20 °C and 20 Hz. *G′* = storage modulus. *G″* = loss modulus. G* = complex modulus. tan *δ* = loss tangent. η* = complex viscosity. *ή* = dynamic viscosity.

**Table 2:** The extent of change in *G*′, *G*″, and *ή* in terms of percentage.

Sample	Time (Day)	*G*′ Change (%)	*G*″ Change (%)	η* Change (%)
Control	1	2.12	−7.67	1.76
	3	38.07	14.62	37.21
	5	77.27	43.53	76.09
5% PPI	1	17.54	−0.46	16.62
	3	23.38	−0.09	22.26
	5	45.05	14.18	43.55
10% PPI	1	−3.86	−12.67	−4.28
	3	4.20	−8.31	3.60
	5	29.46	9.73	28.55
5% SPI	1	11.25	−1.35	10.65
	3	16.04	−2.67	15.20
	5	30.19	6.14	29.11
10% SPI	1	0.61	−11.90	0.12
	3	20.59	1.81	19.84
	5	53.35	24.12	52.22

**Table 3:** Pearson correlation coefficients for various properties of textural and rheological properties.

	Hardness	Springiness	Cohesiveness	Gumminess	Chewiness	Resilience	*G*′	*G*″	G*	tan *δ*	η*	*ή*
Hardness	1.000											
Springiness	0.203	1.000										
Cohesiveness	0.026	0.318 *	1.000									
Gumminess	0.888 **	0.217	0.429 **	1.000								
Chewiness	0.876 **	0.447 **	0.448 **	0.964 **	1.000							
Resilience	0.795 **	0.146	0.431 **	0.938 **	0.871 **	1.000						
*G*′	0.658 **	0.144	0.206	0.680 **	0.659 **	0.692 **	1.000					
*G*″	0.518 **	0.070	0.249	0.578 **	0.539 **	0.619 **	0.973 **	1.000				
G*	0.654 **	0.141	0.208	0.677 **	0.655 **	0.690 **	1.000 **	0.975 **	1.000			
tan *δ*	−0.393 **	−0.214	0.368 *	−0.186	−0.252	−0.069	0.078	0.296 *	0.085	1.000		
η*	0.654 **	0.141	0.208	0.677 **	0.655 **	0.690 **	1.000 **	0.975 **	1.000 **	0.085	1.000	
*ή*	0.517 **	0.070	0.249	0.578 **	0.539 **	0.619 **	0.973 **	1.000 **	0.975 **	0.296	0.975 **	1.000

* Correlation is significant at the 0.05 level (2-tailed). ** Correlation is significant at the 0.01 level (2-tailed).

**Table 4:** Kinetics of starch hydrolysis percentage, hydrolysis index (HI), and estimated glycaemic index (*e*GI) of plain Yangyu jiaotuan (control), with 5% pea protein isolate (5% PPI) and with 10% pea protein isolate (10% PPI) ^a^.

Sample	Time (Day)	C_∞ experimental_ (%)	C_∞ estimated_ (%)	AUC	HI	*e*GI
Control	0	87.13 ± 4.39 ^c^	88.31 ± 3.83 ^de^	9728.93 ± 164.21 ^e^	129.84 ± 2.19 ^e^	110.99 ± 1.20 ^e^
	1st	88.33 ± 4.79 ^c^	87.06 ± 1.30 ^de^	9618.57 ± 288.71 ^e^	128.37 ± 3.85 ^e^	110.18 ± 2.12 ^e^
	3rd	83.83 ± 3.89 ^c^	83.14 ± 6.40 ^cde^	9137.26 ± 411.84 ^de^	121.95 ± 5.50 ^de^	106.66 ± 3.02 ^de^
	5th	78.11 ± 5.13 ^c^	75.46 ± 5.11 ^cd^	8418.05 ± 682.57 ^cde^	112.35 ± 9.11 ^cde^	101.39 ± 5.00 ^cde^
5% PPI	0	85.59 ± 3.97 ^c^	82.35 ± 3.68 ^cde^	9070.40 ± 394.32 ^de^	121.05 ± 5.26 ^de^	106.17 ± 2.89 ^de^
	1st	84.66 ± 2.45 ^c^	81.07 ± 2.06 ^cde^	8922.15 ± 222.79 ^de^	119.08 ± 2.97 ^de^	105.08 ± 1.63 ^de^
	3rd	83.33 ± 0.74 ^c^	79.61 ± 3.19 ^cde^	8679.75 ± 741.24 ^cde^	115.84 ± 9.89 ^cde^	103.31 ± 5.43 ^cde^
	5th	62.09 ± 2.97 ^b^	59.14 ± 3.44 ^b^	6478.45 ± 512.51 ^b^	86.46 ± 6.84 ^b^	87.18 ± 3.76 ^b^
10% PPI	0	82.86 ± 7.47 ^c^	79.01 ± 5.82 ^cde^	8534.99 ± 598.30 ^cde^	113.91 ± 7.98 ^cde^	102.25 ± 4.38 ^cde^
	1st	80.18 ± 5.92 ^c^	76.60 ± 4.70 ^cde^	8257.78 ± 602.94 ^cd^	110.21 ± 8.05 ^cd^	100.21 ± 4.42 ^cd^
	3rd	75.41 ± 5.90 ^c^	70.70 ± 4.91 ^c^	7501.22 ± 507.75 ^c^	100.11 ± 3.72 ^c^	94.67 ± 3.72 ^c^
	5th	45.06 ± 3.43 ^a^	43.06 ± 3.28 ^a^	4822.26 ± 405.86 ^a^	64.36 ± 5.41 ^a^	75.04 ± 2.97 ^a^

Results are expressed as means ± standard deviation (*n* = 3), ^a–e^ values with the same superscript in a column did not differ significantly (*p* < 0.05).

## Data Availability

The data used to support the findings of this study can be made available by the corresponding authors upon request.
